# Characterization of the serine acetyltransferase gene family of *Vitis vinifera* uncovers differences in regulation of OAS synthesis in woody plants

**DOI:** 10.3389/fpls.2015.00074

**Published:** 2015-02-17

**Authors:** Sílvia Tavares, Markus Wirtz, Marcel P. Beier, Jochen Bogs, Rüdiger Hell, Sara Amâncio

**Affiliations:** ^1^Linking Landscape, Environment, Agriculture and Food (LEAF), Instituto Superior de Agronomia, Universidade de LisboaLisbon, Portugal; ^2^Plant Cell Biology Laboratory, Instituto de Tecnologia Química e Biológica, Universidade Nova de LisboaOeiras, Portugal; ^3^Centre for Organismal Studies Heidelberg, University of HeidelbergHeidelberg, Germany; ^4^Studiengang Weinbau und Oenologie, Dienstleistungszentrum Laendlicher Raum RheinpfalzNeustadt, Germany; ^5^Fachbereich 1, Life Sciences and Engineering, Fachhochschule BingenBingen am Rhein, Germany

**Keywords:** OAS (*O*-acetylserine), SERAT, S deficiency, *Vitis vinifera*, drought

## Abstract

In higher plants cysteine biosynthesis is catalyzed by *O*-acetylserine(thiol)lyase (OASTL) and represents the last step of the assimilatory sulfate reduction pathway. It is mainly regulated by provision of *O*-acetylserine (OAS), the nitrogen/carbon containing backbone for fixation of reduced sulfur. OAS is synthesized by Serine acetyltransferase (SERAT), which reversibly interacts with OASTL in the cysteine synthase complex (CSC). In this study we identify and characterize the *SERAT* gene family of the crop plant *Vitis vinifera*. The identified four members of the VvSERAT protein family are assigned to three distinct groups upon their sequence similarities to Arabidopsis SERATs. Expression of fluorescently labeled VvSERAT proteins uncover that the sub-cellular localization of VvSERAT1;1 and VvSERAT3;1 is the cytosol and that VvSERAT2;1 and VvSERAT2;2 localize in addition in plastids and mitochondria, respectively. The purified VvSERATs of group 1 and 2 have higher enzymatic activity than VvSERAT3;1, which display a characteristic C-terminal extension also present in AtSERAT3;1. VvSERAT1;1 and VvSERAT2;2 are evidenced to form the CSC. CSC formation activates VvSERAT2;2, by releasing CSC-associated VvSERAT2;2 from cysteine inhibition. Thus, subcellular distribution of SERAT isoforms and CSC formation in cytosol and mitochondria is conserved between Arabidopsis and grapevine. Surprisingly, VvSERAT2;1 lack the canonical C-terminal tail of plant SERATs, does not form the CSC and is almost insensitive to cysteine inhibition (IC_50_ = 1.9 mM cysteine). Upon sulfate depletion *VvSERAT2;1* is strongly induced at the transcriptional level, while transcription of other *VvSERATs* is almost unaffected in sulfate deprived grapevine cell suspension cultures. Application of abiotic stresses to soil grown grapevine plants revealed isoform-specific induction of *VvSERAT2;1* in leaves upon drought, whereas high light- or temperature- stress hardly trigger *VvSERAT2;1* transcription.

## Introduction

Cysteine biosynthesis is the exclusive entry point of reduced sulfur into primary metabolism of enterobacteria and phototrophic organism. In these organisms, Cys is synthesized from free sulfide and *O*-acetylserine (OAS). The enzyme serine acetyltransferase (SERAT; EC 2.2.1.30) catalyzes the formation of OAS from L-serine and acetyl-coenzyme A (Hell et al., 2002). Further condensation of OAS and sulfide produces cysteine by the action of *O*-acetylserine (thiol) lyase (OASTL, EC 2.5.1.47), an enzyme classified within the β-substituted alanine synthase family (Watanabe et al., 2008a). Sulfur is taken up by all organisms as sulfate, an oxidized form of sulfur that is exclusively reduced to sulfide in plastids of all so far analyzed phototrophic organisms, including green algae, mosses and vascular plants (Shibagaki and Grossman, 2008; Khan et al., 2010). Despite the restriction of sulfide production in plastids, subcellular diversification of the cysteine biosynthesis pathway seemed to take place during the evolution of vascular land plants. While in the unicellular green alga, *Chlamydomonas rheinhardtii*, cysteine biosynthesis is solely restricted to the plastids (Shibagaki and Grossman, 2008), OASTL activity is found in the plastid and in the cytosol of the moss, *Physcomitrella patens* (Birke et al., 2012b), which represents a transition stage from algae to land plants (Lang et al., 2008). Cysteine is an important structural component in many proteins, because of its versatile redox properties. However it also gives rise to presumably hundreds of down-stream metabolites that contain reduced sulfur as functional constituent e.g. iron-sulfur clusters in diverse proteins, vitamins, glucosinolates (Takahashi et al., 2011; Gläser et al., 2014). The most prominent of these sulfur containing low-molecular weight compounds is glutathione, a key player in the oxidative stress response and essential constituent of xenobiotic and heavy metal detoxification pathway found in all eukaryotic cells (reviewed in Noctor et al., 2011). Vascular land plants often suffer from severe abiotic stresses, as a consequence of their sessile lifestyle in an exposed ecological habitat. Thus, cysteine synthesis was believed to be a highly regulated process in all subcellular compartments of land plants. Several lines of evidence demonstrate that cysteine biosynthesis is limited by SERAT activity in vascular plants (Blaszczyk et al., 1999; Wirtz and Hell, 2007; Haas et al., 2008). Accordingly, in *Arabidopsis thaliana* genome SERAT proteins are encoded by a small gene family, whose five members are distributed in the cytosol (AtSERAT1;1, AtSERAT3;1 and AtSERAT3;2), the plastids (AtSERAT2;1) and the mitochondria (AtSERAT2;2) (Kawashima et al., 2005; Watanabe et al., 2008). Functional reverse genomics approaches revealed that either SERAT or OASTL is essential in one of these subcellular compartments (Watanabe et al., 2008; Birke et al., 2013), although each of the OASTL and the SERAT isoforms have defined tasks to maintain cellular cysteine homeostasis (Haas et al., 2008; Watanabe et al., 2010; Birke et al., 2012a; Wirtz et al., 2012). Surprisingly, the activities of both enzymes correlate inversely within these compartments (Haas et al., 2008; Heeg et al., 2008; Watanabe et al., 2008). While most of the OASTL activity is found in cytosol (~50%, OASTL-A, At4g14880) and plastids (~45%, OASTL-B, At2g43750) of Arabidopsis, mitochondrial OASTL-C (At3g59760) contributes only 5% to total foliar OASTL activity (Heeg et al., 2008; Birke et al., 2013). In contrast, mitochondrial AtSERAT2;2 (At3g13110) is the pacemaker of cysteine synthesis and contributes in leaves approximately 80% of total SERAT activity, while cytosolic AtSERAT1;1 (At5g56760) and plastidic AtSERAT2;1 (At1g55920) amount to 15 and 5% of the remaining total SERAT activity, respectively (Haas et al., 2008; Watanabe et al., 2008). The insignificant contribution to extractable total SERAT activity defines SERATs of the group 3 as minor SERAT isoforms in Arabidopsis, which is in full agreement with low transcription of *AtSERAT3;1* (At2g17640) and *AtSERAT3;2* (At4g35640) and poor enzymatic activities of recombinant AtSERAT3;1 and AtSERAT3;2 proteins, when compared to the major SERAT belonging to group 1 and 2 (Kawashima et al., 2005). Consequently, quadruple *SERAT* loss-of-function mutants lacking all major SERATs display a strongly retarded growth phenotype (Watanabe et al., 2008). The inability to interact with OASTL in the cysteine synthase complex is another feature, which separates the minor SERATs of group 3 from major SERATs (Francois et al., 2006; Yi et al., 2013). This interaction has been demonstrated to activate AtSERAT2;2 by releasing it from cysteine inhibition, which is an important determinant of SERAT activity in all subcellular compartments (Wirtz et al., 2012). *SERAT* genes belonging to group 1 and 2 are not transcriptionally regulated upon sulfur deficiency and the total SERAT activity in Arabidopsis is hardly affected by depletion of sulfate in the environment (Kawashima et al., 2005; reviewed in Takahashi et al., 2011). It is therefore accepted that SERAT activity in Arabidopsis is mainly regulated at the post-translational level by CSC formation and cysteine feedback-inhibition of SERAT activity (Noji et al., 1998). This is a fundamental difference to the regulation of sulfide production in plastids. In Arabidopsis, transcription of high affinity sulfate transporters and sulfate reducing enzymes (e.g., adenosine-5′-phosphosulfate reductase) is subject of extensive regulation in response to sulfate supply, internal cysteine demand for primary and secondary metabolism and growth stimuli like nitrogen supply and light (Rouached et al., 2008; Mugford et al., 2009; Davidian and Kopriva, 2010; Takahashi et al., 2011).

The presence of SERAT and OASTL isoforms in sub-celular compartments with own protein-biosynthesis along with the unequal distribution of their activities in the cytosol, the plastids and the mitochondria, seem to be conserved among vascular plants, e.g., *Brassica oleracea, Datura innoxia* (Rolland et al., 1992; Kuske et al., 1996) and *Pisum sativum* (Ruffet et al., 1995). Surprisingly, purified mitochondrial extracts from spinach (*Spinacea oleracea*) lack SERAT activity (Brunold and Suter, 1982) and it remains uncertain whether a true OASTL is present in mitochondria. Although mitochondrial OASTL activity could be detected in spinach (Lunn et al., 1990), it was later identified to be a side activity of β-cyanoalanine synthase (Warrilow and Hawkesford, 2000). Taken together, these results strongly indicate that there is no mitochondrial CSC in spinach and the moss *P. patens*, which questions the importance of CSC formation for regulation of cellular cysteine synthesis in land plants.

Tavares et al. (2008) identified *Vitis vinifera* sulfate transporters upon the release of grapevine genome (Jaillon et al., 2007; Velasco et al., 2007). The link between sulfate deficiency and grapevine secondary metabolism was investigated and the increased of phenolic and stilbene compounds was observed in grapevine cells and plantlets (Tavares et al., 2013). In this study we addressed for the first time the importance of cysteine biosynthesis in different sub-cellular compartments of vascular land plants by characterization of the SERAT protein family of grapevine. We select grapevine for this comparative approach, since grapevine is a perennial plant and does not produce glucosinolates, which contribute significantly to the high amount of total sulfur found in the annual model plant Arabidopsis (reviewed in Khan et al., 2010).

## Results

### Identification of serine acetyltransferases in *Vitis vinifera*

The so far best characterized plant species with respect to structure function relationship of SERAT proteins, *Arabidopsis thaliana* (Brassicaceae) and *Glycine max* (Fabaceae), separate about 115 million years from *Vitis vinifera* (Vitaceae) (Wikström et al., 2001). Thus, we used a degenerated primer approach to amplify putative SERAT sequences from a grapevine leaf cDNA. Degenerate primers were designed to a consensus plant SERAT sequence obtained by aligning all nucleotide sequences from verified *SERAT* genes deposited in NCBI. This approach revealed nine putative VvSERAT protein sequences that all contain the canonical hexapeptide transferase motitf (IPR001451) and display high homology (<e^−10^) to a translated fragment of VvSERAT (ABY86367.1). A detailed alignment analysis decreased the number of putative candidates from nine to four, since some sequences were redundant arising from both projects responsible for grapevine genome sequencing. The four sequences cluster after consistency-based multiple sequence alignment (T-Coffee, EMBL, Figure S1) in the three distinct SERATs groups defined in Watanabe et al. (2008). According to the grouping in these clusters the four putative SERATs were re-named VvSERAT1;1 (following the Arabidopsis nomenclature that was developed by associating the *VvSERAT* to the *AtSERAT* genes, S1), VvSERAT2;1, VvSERAT2;2 and VvSERAT3;1. The native 5′- and 3′-ends of mature VvSERAT2;1 and VvSERAT2;2 mRNAs were experimentally verified by RACE. The sequences of mature spliced mRNAs and the corresponding translated full length proteins of *V. vinifera* cv Touriga Nacional corresponded to the NCBI accessions; XM_002282514/XP_002282550 for VvSERAT1;1 and XM_002270508/XP_002270544 for VvSERAT2;1 (Supplementary Table 1). VvSERAT2;2 and VvSERA3;1 displayed several discrepancies (Figure S2) to the sequences in NCBI database resulting from *V. vinifera* genome sequencing, and both sequences were deposited at NCBI with the accession numbers of KP074964 and KP074965, respectively. The identification of the *VvSERATs* genes, as well as the characterization of DNA structure can be consulted in Supplementary Table 1 and Figure S3.

**Figure 1:**
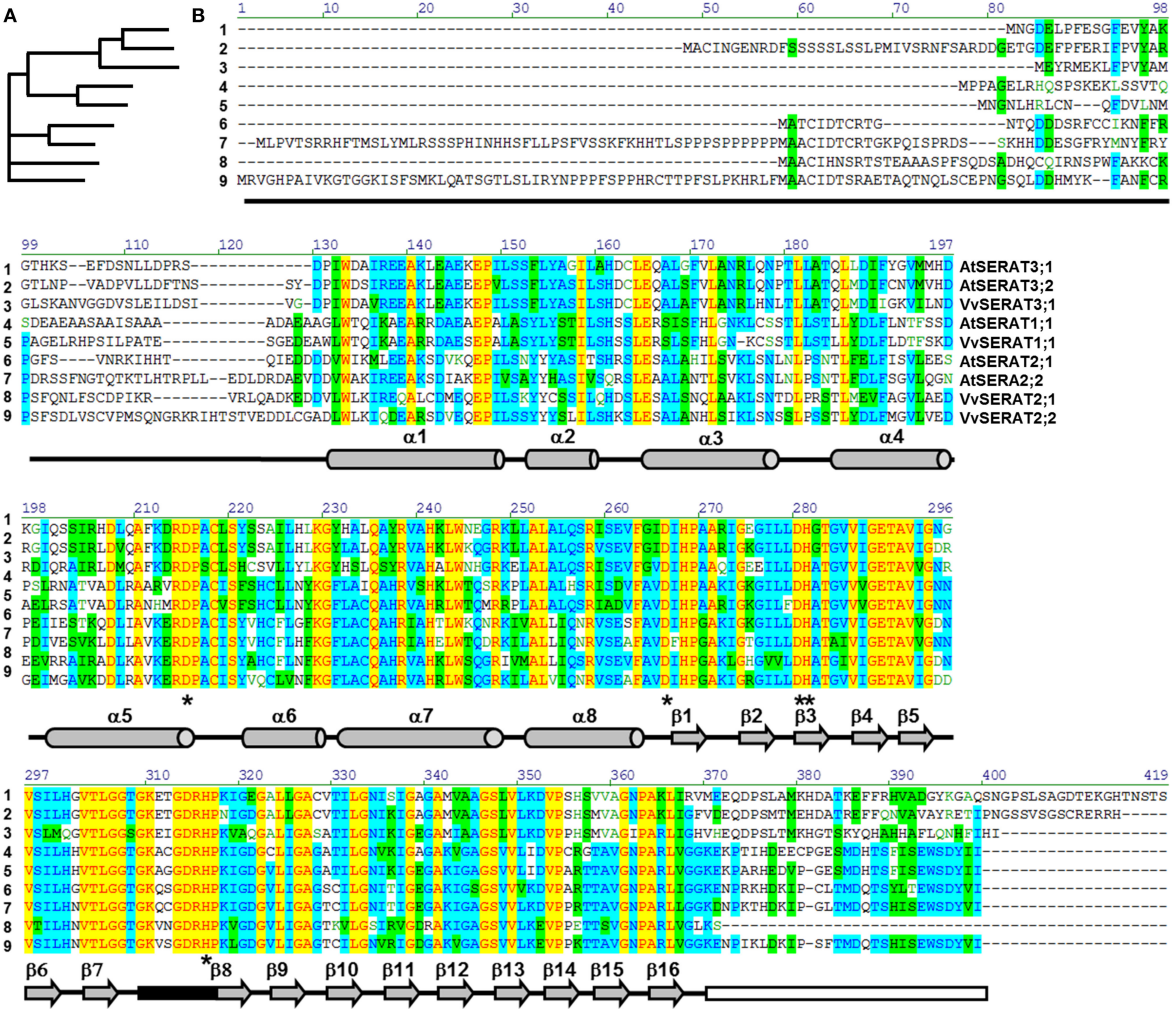
**Comparison of SERAT protein families in grapevine and Arabidopsis**. Full length protein sequences of biochemically verified SERAT proteins from Arabidopsis and grapevine were compared with the AlignX tool of the Vector NTI Advance 9.1.0 software suite (Invitrogen, Germany). **(A)**, Guide tree. **(B)**, Protein-sequence alignment, Color code indicates degree of similarity (yellow, font red: identical; cyan, font blue: conservative; green, font black: block of similar; no color, font green: weakly similar, no color, font black: non-similar). Secondary structural elements are shown below the sequences: gray barrels, α-helices, gray arrows, β-sheets, black box, active site, white box, C-terminal tail of major SERATs (group 1 and 2) that mediates interaction with OASTL, asterisks indicate residues known to be important for enzymatic activity.

The alignment of VvSERATs with AtSERATs demonstrates a high similarity of SERAT proteins (Figure 1) from both species within the three groups. As expected group 3 SERATs from *V. vinifera* and *A. thaliana* are characterized by an extension at the C-terminus, when compared to SERATs of group 1 and 2 (Figure 1). This extension is supposed to inhibit the interaction of group 3 SERATs with OASTLs (Mino et al., 2000; Francois et al., 2006). Since SERATs of group 3 from Arabidopsis display minor catalytic activity and are supposed to function *in vivo* as acetyltransferases that address so far unknown substrates, the subsequent analyses mainly focuses on VvSERAT1;1, VvSERAT2;1 and VvSERAT2;2. The canonical α-helical N-terminus as well as the C-terminal β-sheet structure of SERAT monomers is conserved in these VvSERATs (Figure 1). The latter is followed in the AtSERATs of group 1 and 2 by a highly conserved C-terminal tail (EWSDY(V/I)I), which promotes the interaction of SERATs with OASTLs in bacteria and plants and is mandatory for the inhibition of SERATs by cysteine (Olsen et al., 2004; Francois et al., 2006; Feldman-Salit et al., 2009; Wirtz et al., 2010). Surprisingly, this C-terminal tail is specifically deleted in VvSERAT2;1, while VvSERAT1;1 and VvSERAT2;2 have C-terminal tails that match the canonical OASTL interaction motif (Figure 1).

### Subcellular localization of the serat protein family in grapevine

The sub-cellular localization of the identified full-length VvSERATs was predicted *in silico* with the algorithm of the MultiLoc server (http://www-bs.informatik.uni-tuebingen.de/Services/MultiLoc/index_html). In agreement with the clustering according to sequence alignments, VvSERAT1;1 and VvSERAT3;1 were predicted to be cytosolic (probability scores: 0.92 and, 0.95, respectively). The VvSERAT2;2 showed a very significant score for localization in mitochondria (score 0.85), while VvSERAT2;1 was predicted to localize in the cytoplasm (probability score 0.63) and/or with lower score (probability score 0.33) to the chloroplast. We confirmed the prediction of plastidic VvSERAT2;1 localization with TargetP1.1 (http://www.cbs.dtu.dk/services/TargetP/) and ChloroP1-1 (http://www.cbs.dtu.dk/services/ChloroP/) that both provide also high probability scores for plastidic localization of VvSERAT2;1 (0.66 and 0.48, respectively) and predicted a transit peptide of 57 amino acids.

**Figure 2:**
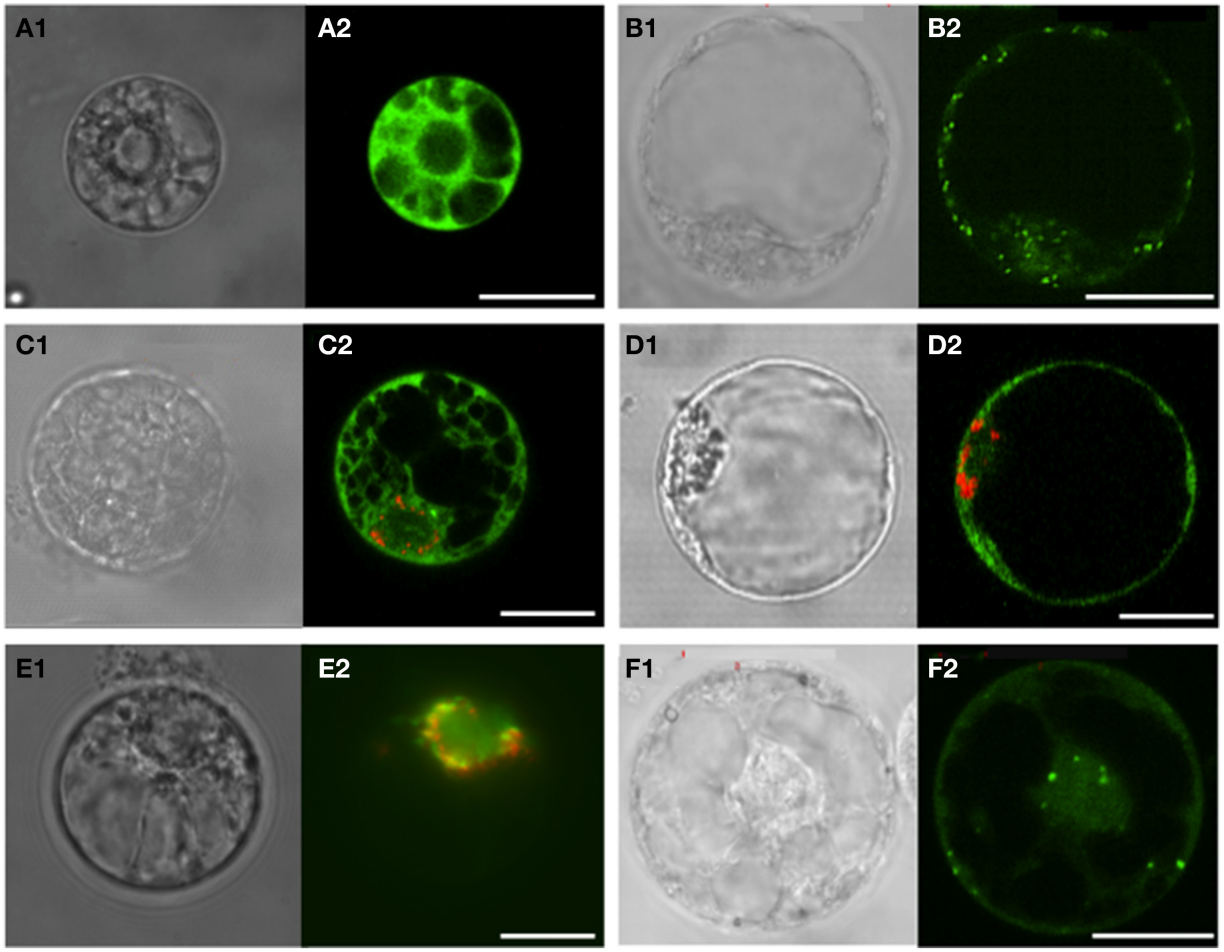
**Sub-cellular localization of GFP fused to the C-terminal sequences of *Vitis vinifera* cv Touriga Nacional SERAT proteins**. Electroporation was carried out in *V. vinifera* protoplasts isolated from cell cultures. The signal expression and localization of GFP and RFP was observed between 24 and 48 h incubation by confocal laser scanning or epifluorescence microscopy. **(A)** Plasmid pFF19-GFP used as control for localization into the cytosol; **(B)** SHMT-GFP carrying the transit peptide of Serine hydroxymethyltransferase (SHMT) from Arabidopsis as a control for mitochondria targeting; **(C,D)** VvSERAT1;1-GFP and VvSERAT3;1-GFP co-transformed with VsRSS:RFP carrying the transit peptide of pea Rubisco small subunit as a control for plastidic localization, respectively. Both VvSERAT isoforms show cytosolic localization; **(E)** VvSERAT2;1-GFP and VsRSS:RFP co-localizing in the plastids; **(F)** VvSERAT2;2-GFP localized In the mitochondria and the cytosol. Letters followed by 1, Protoplasts observed in contrast phase microscopy; Letters followed by 2, Confocal laser scanning except for E2 observed by epifluorescence microscopy. Scale bars = 20 μm.

Since the *in silico* analysis of VvSERATs localization was not clear without ambiguity, the subcellular localization of VvSERAT fused via the C-terminus to the green fluorescent protein (GFP) was determined in *V. vinifera* protoplasts. As sub-cellular localization marker, we used the untagged GFP (pFF19-GFP, Figure 2A) for localization in the cytosol, GFP fused to transit peptide of Arabidopsis Serine hydroxymethyltransferase (SHMT) for mitochondrial localization (AtSHMT:GFP, Figure 2B) and the red fluorescent protein (RFP) in fusion with the transit peptide of pea Rubisco small subunit (VsRSS:RFP, Figures 2C,D,E) for localization in the plastids. Ectopic expression of the candidate and the marker GFP-fusion protein demonstrated the cytosolic localization of the VvSERAT1;1 (Figure 2C) and VvSERAT3;1 GFP fusion proteins (Figure 2D). In order to avoid aggregation of VvSERAT2;1:GFP a low concentration of plasmid was used for electroporation of protoplast, which results in only faint signal for VvSERAT2;1:GFP. However this signal co-localized with the plastid-targeted VsRSS:RFP signal. A note of caution must be added here since the VsRSS:RFP protein was also found in the cytosol of transformed Vitis protoplasts isolated from cell culture (Figure 2E). As predicted by the analysis of the putative mitochondrial transit peptide of VvSERAT2;2, the signal of VvSERAT2;2:GFP (Figure 2F) was almost identical to the signal distribution of protoplasts transformed with AtSHMT:GFP (Figure 2B). These results indicate that VvSERAT2;1 and VvSERAT2;2 are the *in organello* localized isoforms, which is in agreement with the subcellular localization of the homologous proteins in Arabidopsis. The dual targeting of these isoforms in the cytosol and the plastids or mitochondria is presumably an artifact of the ectopic expression of the VvSERAT2:GFP fusion proteins in grapevine protoplast, since also *bona fide* marker proteins for mitochondrial or plastidic localization were partly found in the cytosol in this system after ectopic expression driven by the 35S-promotor (Figures 2D,F).

### Biochemical characterization of the serat protein family of grapevine

**Figure 3:**
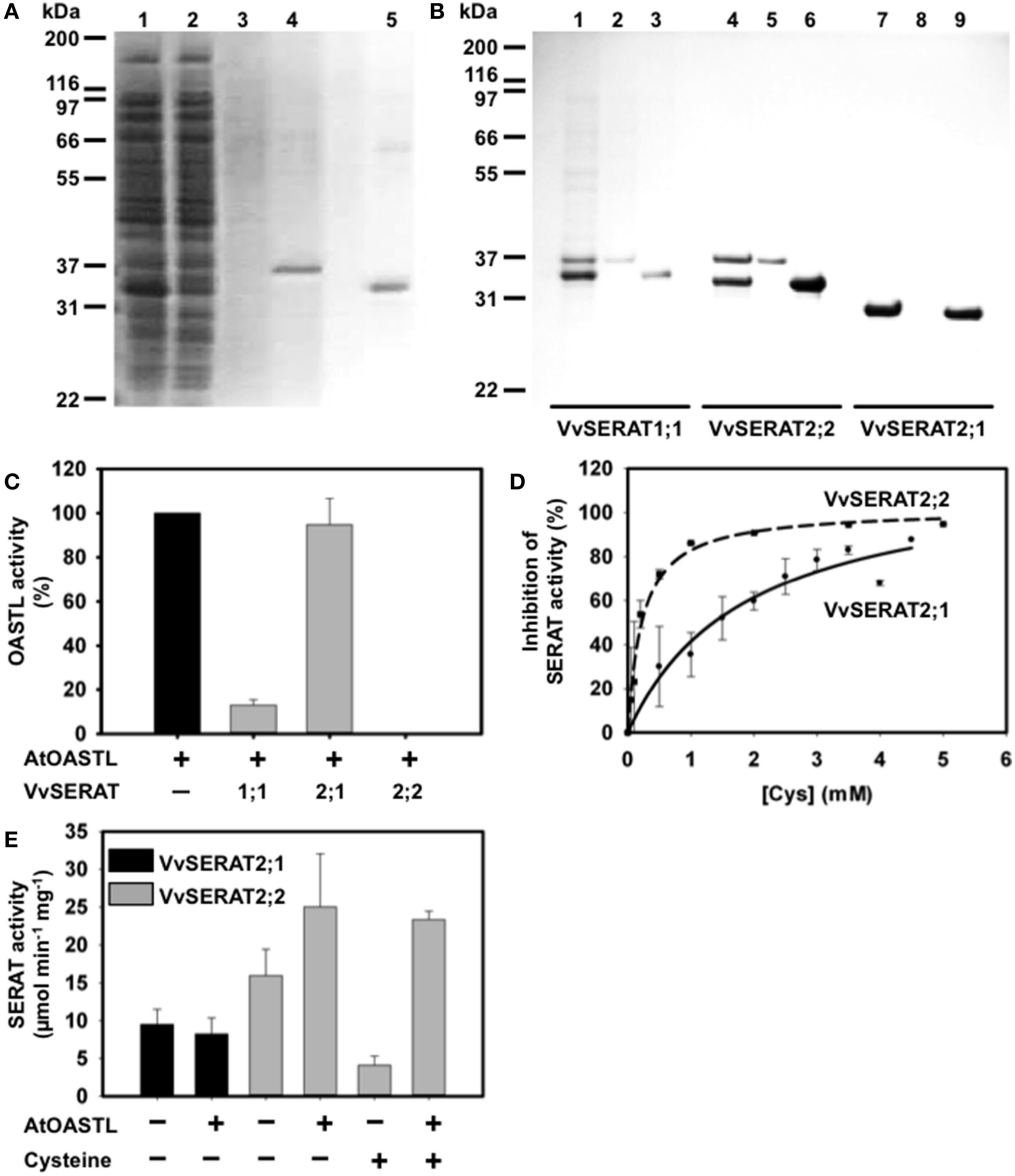
**Biochemical characterization of the SERAT protein family of grapevine**. **(A)** Purification of recombinant VvSERAT2;2 in fusion with a N-terminal His tag and immobilized in a metal affinity of column. SDS-PAGE gel stained with Coomassie blue. 1, *E. coli* crude proteins after expression of VvSERAT2;2 protein; 2, flow through after the contact with Ni affinity column; 3, washing steps with extraction buffer containing 10 mM OAS and 80 mM imidazole; 4, elution of AtOASTL-B with extraction buffer containing 400 mM imidazole; 5, elution of VvSERAT2;2 with extraction buffer containing 400 mM imidazole. **(B)** Interaction between AtOASTL-B and VvSERAT and the formation of the cysteine synthase complex (CSC). SDS-PAGE gel stained with Coomassie blue. Elution from the Ni affinity column: 1, 4, 7—CSC elution with extraction buffer supplemented with 10 mM OAS; 2, 5, 8—AtOASTL-B elution with 400 mM imidazole in extraction buffer; 3, 6, 9—VvSERAT elution using 400 mM imidazole. 1, 2 and 3—VvSERAT1;1; 4, 5 and 6—VvSERAT 2; 2, 7, 8 and 9—VvSERAT2;1. **(C)** Inhibition of AtOASTL-B activity by addition of VvSERATs. AtOASTL-B varied between 0.2 and 1 pmol and was incubated in absence (control) or presence of four-fold excess of VvSERATs to allow CSC formation. **(D)** Feedback inhibition of recombinant VvSERAT2;1 and VvSERAT2;2 by L-Cys. Assays were carried out as described in “Material and Methods” section. IC50 determined at 1.9 mM for VvSERAT2;1 and at 0.188 mM for VvSERAT2;2. E, Serine acetyltransferase activity (SERAT) of recombinant VvSERAT2;1 and 2;2 purified fractions, in the absence (−) or the presence (+) of AtOASTL-B (0.8 μg) and of cysteine (0.14 mM).

The cDNA encoding for full length VvSERAT1;1 and VvSERAT2;1 and SERAT2;2 lacking the predicted transit peptide were amplified with primers containing a *Bam*HI/*Xho*I restriction endonuclease site (Supplemental Table 2) and cloned in the expression vector pET28a (Invitrogen, Germany). The resulting constructs allowed ectopic expression of VvSERATs in fusion with an N-terminally located His tag in *E. coli*. Immobilized metal affinity purification of VvSERAT1;1, VvSERAT2;1 (Figure 3) and VvSERAT2;2 resulted in apparently pure VvSERAT proteins as demonstrated by Coomassie staining of SDS-PAGE separated VvSERAT fractions (Figures 3A,B). In all cases the apparent molecular weight of the purified recombinant fusion proteins was in agreement with the theoretically determined molecular weight of the His-VvSERAT fusion proteins. All His-VvSERAT proteins displayed significant enzymatic SERAT activity, which demonstrates the identity of these candidate proteins as true SERATs (Table 1). VvSERAT2;2 was sensitive to feedback inhibition by cysteine concentrations (Table 1) that were determined in leaves of higher plants (Krüger et al., 2009). In contrast VvSERAT2;1, which lacks the conserved C-terminal tail, was ten-times less sensitive toward cysteine than the mitochondrial VvSERAT2;2 (Figure 3D, Table 1), although the remaining structural core elements and the active site were highly conserved between VvSERAT2;1 and VvSERAT2;2 (Figure 1). Since the C-terminal tail of AtSERAT2;2 is a prerequisite for CSC formation and CSC formation regulates cysteine feedback-sensitivity of SERAT2;2 in Arabidopsis, we tested the ability of purified His-VvSERATs to bind recombinant OASTL from Arabidopsis (AtOASTL-B). Only VvSERAT1;1 and VvSERAT2;2 were able to form a heterologous CSC with AtOASTL-B, which was dissociable by addition of OAS (Figure 3B). Furthermore, titration of OASTL B with these VvSERAT isoforms resulted in strong inhibition of OASTL activity, which is in agreement with binding of the SERAT C-terminal tail in the active site of OASTL during CSC formation. VvSERAT2;1 lacks the canonical C-terminal tail and was, thus, unable to repress OASTL activity after titration (Figure 3C). In order to dissect the function of potential CSC formation in organelles of grapevine, we compared the regulatory impact of OASTL on group 2 VvSERATs *in vitro*. As expected addition of OASTL to VvSERAT2;1 had no significant impact on SERAT activity. In contrast, CSC formation increased enzymatic activity of VvSERAT2;2 almost two-fold and released VvSERAT2;2 from cysteine inhibition (Figure 3E, Table 1).

**Table 1:** **Subcellular localization and biochemical properties of SERAT proteins in grapevine**.

**Isoform name**	**Subcellular Localization**	**MW (kDa)**	**SERAT activity (μmol min^−1^ mg^−1^)**	**Cys Inhibition (IC_50_ mM)**	**Interaction with OASTL**	**SERAT activity in CSC (μmol min^−1^ mg^−1^)**
VvSERAT1;1	C	32.34	0.94 ± 0.36	n. d.	+	n. d.
VvSERAT2;1	P, < C	29.46	9.6 ± 2	1.9 ± 0.56	−	n. a.
VvSERAT2;2	M, < C	32.38	16 ± 3.5	0.16 ± 0.03	+	25 ± 7
VvSERAT3;1	C	n.d.	0.13 ± 0.01	n. d.	n. d.	n. d.

*Subcellular localization of VvSERATs has been tested by ectopic expression of VvSERAT:GFP fusion proteins in grapevine protoplasts (Figure 2). C, cytosol, P, plastids, M, mitochondrion. Theoretical molecular weight (MW) was determined for the His-VvSERAT fusion proteins. Purified recombinant His-tagged VvSERATs were tested in presence or absence of 5-molar excess of AtOASTL-B for enzymatic activity according to Wirtz et al. (2001). The inhibition constant for cysteine (IC_50_) was determined by titration of free VvSERATs with up to 5 mM cysteine (Figure 3). Interaction of VvSERAT with OASTL has been demonstrated by inhibition of OASTL activity upon CSC formation (Figure 3). (N = 3−5, n. a., not applicable, n.d., not determined)*.

### Impact of sulfate supply on regulation of cysteine synthesis

**Figure 4:**
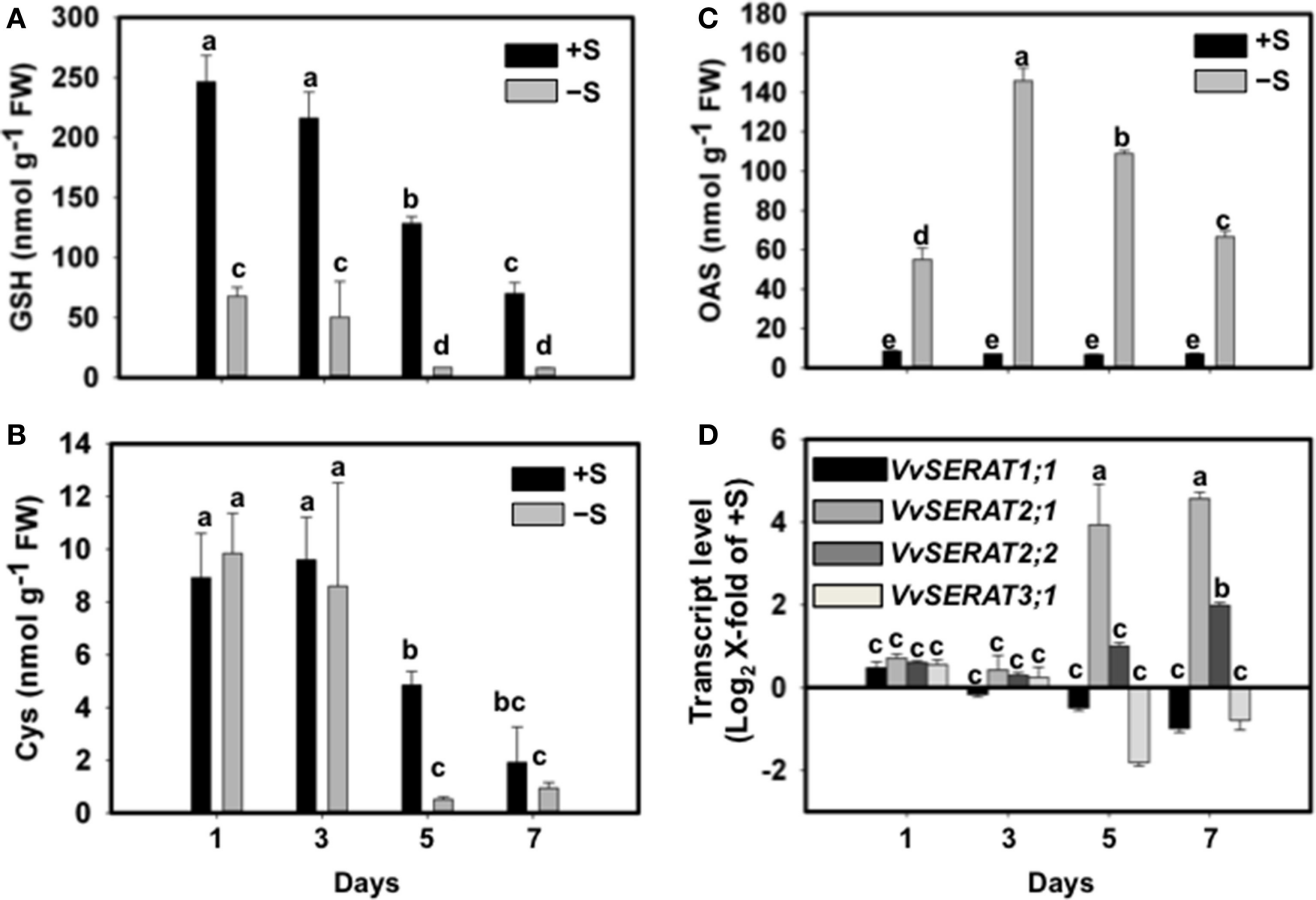
**Effect of sulfate supply on the regulation of cysteine synthesis in Vitis vinifera cv Touriga Nacional cell suspensions**. Thiols; GSH **(A)**, cysteine **(B)**, and OAS **(C)** content was analyzed in sufficient (+S) and deficient sulfate cell cultures (−S) and media. **(D)** Transcript levels of *VvSERAT* genes were determined by RT-qPCR from the +S and −S cells. From cell suspensions grown in −S (50 μM SO^2−^_4_) or +S (1.5 mM SO^2−^_4_) medium, samples were taken at day 1, 3, 5 and 7 of growth. cDNA obtained from RNA extracted from cell suspensions was normalized against the expression of *Act2* RNA as described in Methods. The raw Ct values for *Act2* expression in *V. vinifera* cells were 18.3 and 19.1 at day1, 18.1 and 17.8 at day 3, 18.9 and 18.2 at day 5, and 19.6 and 19.1 at day 7, respectively under −S and +S conditions. Error bars represent ± SD; *n* = 6. Different letters indicate statistically significant differences using Two-Away ANOVA (*p* < 0.05).

Sulfate is exclusively reduced to sulfide in plastids of higher plants (Davidian and Kopriva, 2010). The absence of CSC formation in plastids of *V. vinifera* prompted us to test how cysteine synthesis is regulated in grapevine cells upon sulfate depletion. We thus challenged a grapevine suspension cell culture with sub-optimal sulfate levels (50 μM SO^2−^_4_) for up to 7 days. The grapevine cells adopt within 1 day, under a suboptimal sulfate supply, a >4-fold decrease of the glutathione steady state level (Figure 4A). This adopted level of glutathione was kept until 3 days of sub-optimal supply. Until this time point the cysteine level was unaffected when compared to cells grown on optimal sulfate supply (1.5 mM SO^2−^_4_, Figure 4B). At later time points (day 5–7) cysteine and glutathione levels drop dramatically in cells grown on 50 μM sulfate (Figures 4A,B), indicating that exogenous sulfate was used up and its lack limited cells growth. Interestingly, the steady state levels of the cysteine pre-cursor, OAS, displayed a different pattern upon sulfate depletion: OAS level increase 5-fold upon external sulfate depletion within 1 day and reached a maximum at day three. Prolonged sulfate limitation (day 5–7) caused decrease of OAS to levels of day one of sulfate depletion (Figure 4C). Under optimal sulfate supply the cellular OAS level remained unaffected during the entire period of observation.

Sub-optimal sulfate supply for up to 3 days did not affect transcription of the here identified VvSERATs. However, prolonged sulfate depletion, which was accompanied with decreased cysteine levels, caused 3.9-fold (Log_2_X-fold of +S) increase of *VvSERAT2;1* mRNA steady state levels at day five, which increase even more on day seven (4.5-fold, Figure 4D). The other *VvSERAT* genes were not regulated at the transcriptional level in response to sulfate availability, with the exception of *VvSERAT2;2* that was slightly induced only at day seven of sulfate deprivation (Figure 4D).

### Impact of environmental stresses on *SERAT* transcription

**Figure 5:**
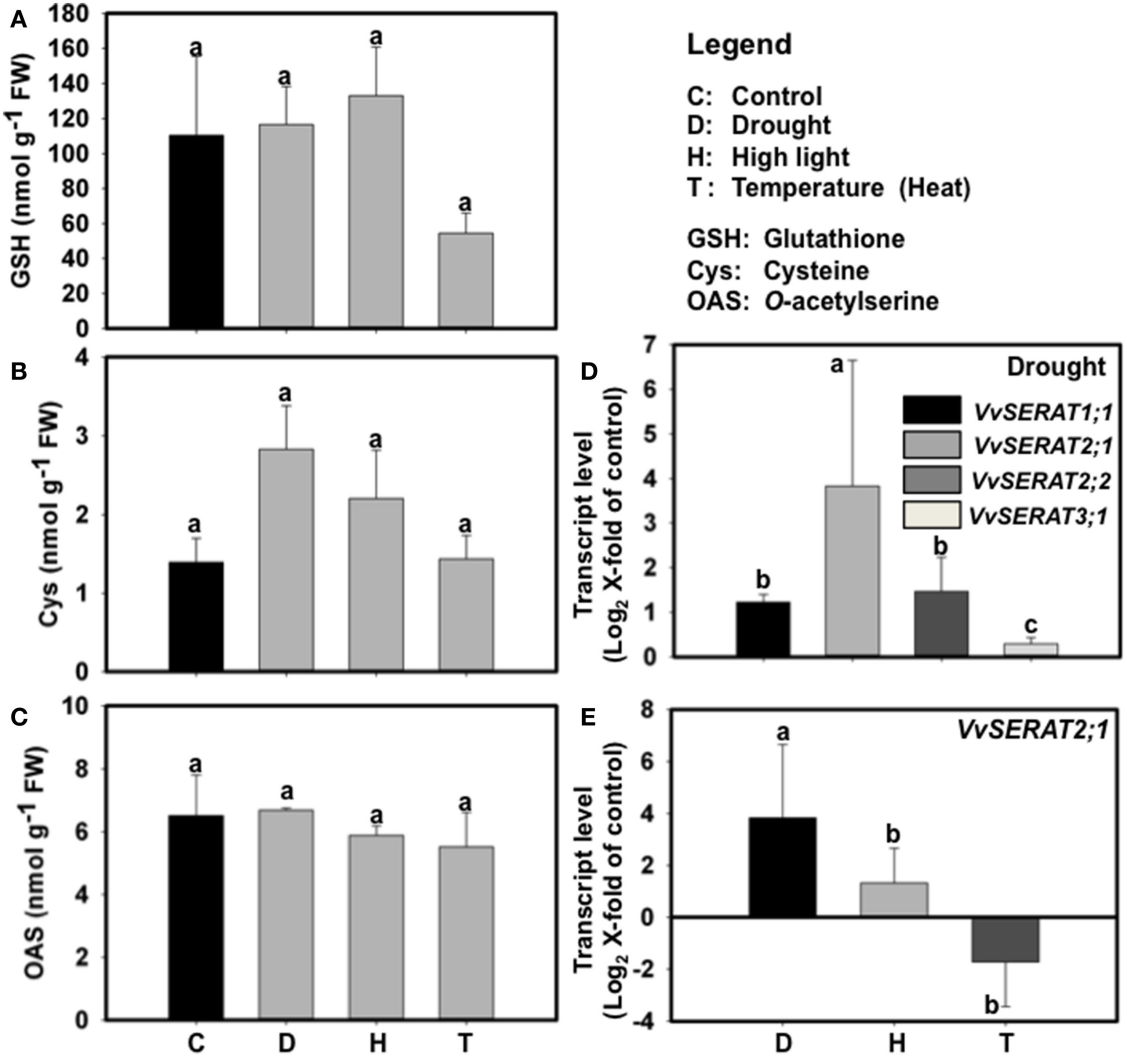
**Effect of abiotic stresses on the contents of thiols (GSH, A and cysteine, B) and OAS (C), and *VvSERAT* transcripts level in leaves of *V. vinifera* cv Touriga Nacional (E, D)**. D—Drought, H—High light, T—Heat Stress. cDNA obtained from leaves RNA was normalized against the expression of three reference genes as described in Coito et al. (2012). Error bars represent ±SD; *n* = 6. Statistically significant differences were identified using One-Away ANOVA (*p* < 0.05), different letters indicate statistically significant differences.

The unexpected strong transcriptional regulation of *VvSERAT2;1* in response to suboptimal sulfate supply indicated that *VvSERAT2;1* expression is sensitive to abiotic stresses. We therefore tested if environmental factors that mostly affect growth performance of grapevine in its natural habitat (drought, high-light and temperature) cause regulation of cysteine biosynthesis. These abiotic stresses are known to affect glutathione turnover in higher plants and are consequently supposed to disturb cysteine- and glutathione-biosynthesis (Noctor et al., 2011). However, only application of heat stress caused a significant down-regulation of glutathione steady state levels in grapevine leaves (Figure 5A), while the cysteine steady state level was unaffected (Figure 5B). Surprisingly, OAS levels remained unaffected in grapevine leaves upon drought, high light- and heat-stress (Figure 5C). The only stress condition that significantly disturbs cysteine homeostasis was water deficiency, which resulted in an approximately two-fold up-regulation of foliar cysteine steady state level (Figure 5B). We thus tested transcription of all identified *VvSERATs* under drought stress in leaves. Drought stress did not affect transcription of *VvSERAT3;1* and resulted in statistically significant but weak up-regulation of *VvSERAT1;1* (~1.2-fold) and *VvSERAT2;2* (<1.6-fold) genes, which biological relevance might be questionable (Figure 5D). In contrast, transcription of *VvSERAT2;1* was 3.8-fold induced by water deficiency and might be the trigger for up-regulated cysteine steady state levels upon this abiotic stress (Figure 5D). Other here tested abiotic stresses (heat and high-light stress) did not cause an increase in *VvSERAT2;1* mRNA level strongly indicating that the up-regulation of *VvSERAT2;1* transcription is a specific response to drought and not a consequence of pleiotropic stress signaling (Figure 5E).

## Discussion

### Conservation of the SERAT protein family in higher plants

Phylogenetic analysis of the SERAT amino acids sequence strongly indicated that in Arabidopsis the ancestral SERAT gene is of host origin and is not derived of the cyanobacterial endosymbiont (Kopriva et al., 2008). Thus, diversification of the SERAT gene family during evolution of higher plants presumably starts with identical nuclear encoded feedstock. A significant conservation of the SERAT gene family in vascular land plants at the genomic level was already indicated by earlier comparison of sequenced genomes for different model plants e.g., Arabidopsis, soybean and rice (Watanabe et al., 2008). The identification and biochemical characterization of four SERAT proteins in grapevine provides functional confirmation that the encoded VvSERAT proteins share indeed many biochemical properties and sub-cellular localization with their homologous Arabidopsis proteins (Kawashima et al., 2005). This is in agreement with most of the previous studies on SERAT activity distribution in sub-cellular fractions except in case of spinach, which seems to lack mitochondrial isoforms SERAT and OASTL (Brunold and Suter, 1982; Warrilow and Hawkesford, 2000).

The humble enzymatic activity of VvSERAT3;1 unravel that, like in Arabidopsis (Kawashima et al., 2005), the SERATs belonging to group 1 and 2 contribute the bulk of SERAT activity in grapevine under regular sulfate supply. Only transcription of the group 3 SERATs is induced upon sulfate deficiency in roots of Arabidopsis (Kawashima et al., 2005). However, VvSERAT3;1 is not transcriptionally regulated by sulfate depletion or in response to drought stress, which questions a significant role of SERAT3;1 in grapevine upon stresses that require increase of cysteine production.

A major trigger for regulation of cellular SERAT activity in plants is feedback inhibition by cysteine (Takagi et al., 1999b). In Arabidopsis the major isoforms of SERATs differ in their degree of cysteine inhibition, which allows fine tuning of OAS synthesis in each sub-cellular compartment (Noji et al., 1998). In grapevine, the feedback sensitivity of the VvSERAT2;1 is ten-times lower than that of VvSERAT2;2 and cytosolic VvSERAT1;1. The significant difference between two *in organello* localized VvSERATs is most likely due to deletion of VvSERAT2;1 C-terminus, which eliminates the methionine at position 256, a residue known to determine cysteine sensitivity (Inoue et al., 1999). Biochemical characterization of plastid-localized SERATs from spinach and Arabidopsis uncovers very low conservation of SERAT cysteine sensitivity in plastids. While the plastidic isoform of spinach is fully inhibited by 0.1 mM cysteine (Noji et al., 2001), AtSERAT2;1 is almost not affected by this cysteine concentration (Noji et al., 1998). Interestingly, a tobacco SERAT belonging to group 2 is similarly cysteine feedback insensitive than VvSERAT2;1 and possess also a 3 amino acids deletion in the C-terminal tail. As a result of its diminished feedback-sensitivity the tobacco SERAT was successfully applied for biotechnological production of cysteine in *E. coli* (Wirtz and Hell, 2003), since SERAT activity (CysE, IC_50_ ≈1 μM cysteine) in enterobacteria is fully inhibited even by low cysteine levels to avoid induction of the Cys-operon. This strong cysteine inhibition is mediated by the C-terminal tail of CysE that blocks binding of the pantentheinyl arm of acetyl-CoA to the active site of CysE when cysteine is present (Takagi et al., 1999a; Olsen et al., 2004).

### Regulation of SERAT activity by cysteine inhibition in mitochondria

The cysteine dependent competition of the SERAT C-terminal tail with acetyl-CoA for binding to the active site of SERAT, presumably, provides the structural basis for regulation of VvSERAT2;2 activity upon CSC formation, since reorientation of the SERAT C-terminal tail is supposed to be a prerequisite for interaction of SERAT and OASTL (Francois et al., 2006; Wirtz et al., 2010; Feldman-Salit et al., 2012). Indeed formation of a heterologous CSC with OASTL from Arabidopsis releases recombinant VvSERAT2;2 from cysteine inhibition. In Arabidopsis cysteine-feedback sensitivity of AtSERAT2;2 is also controlled by association status in the mitochondrial CSC (Wirtz et al., 2012). Since CSC formation is dependent on sulfide and OAS steady state levels (Wirtz and Hell, 2006), mitochondrial SERAT activity in grapevine and Arabidopsis is not only controlled by cysteine but also by supply of OAS and sulfide. The latter allows regulation of SERAT activity in response to carbon/nitrogen and sulfur supply in both plant species and probably reflects a concept that is used by higher plants also in other sub-cellular compartments, e.g., the cytosolic SERAT of soybean is also released from cysteine inhibition by CSC formation (Kumaran et al., 2009; Hell and Wirtz, 2011; Takahashi et al., 2011; Jez and Dey, 2013). However, recombinant AtSERAT1;1 was only marginally activated by CSC formation, indicating that cysteine feedback sensitivity of SERATs contributes with varying degree to regulation of OAS synthesis in different plant species (Wirtz et al., 2012).

### Regulation of SERAT activity in chloroplasts

Interaction of SERATs with OASTL to form the CSC was supposed to be a hallmark of bacterial SERATs and the major plant SERATs belonging to group 1 and 2, which all display high SERAT activity when compared to SERATs of group 3 (this work, Kawashima et al., 2005). Only in the enteric protozoan parasite *Entamoeba histolytica* CSC formation by the major SERAT is absent as a result of an atypical structural alteration in the C-terminus of EhSERAT (Kumar et al., 2011). The EhSERAT is furthermore characterized by a very distinct N-terminal α–helical domain, which prevents dimerization of the SERAT trimers. This dimerization is another canonical structural feature of plant (Feldman-Salit et al., 2009; Wirtz et al., 2010) and bacterial SERATs (Hindson et al., 2000) under non-stressed conditions (Mino et al., 2001). Accordingly, the cysteine synthesis machinery seems to operate differently in *E. histolytica* when compared to plants, which is most likely due to the different life style of these eukaryotes. The identification of a plant SERAT belonging to group 2 that, like EhSERAT, most certainly lacks the ability to form the CSC is thus remarkable and surprising. Four lines of evidence demonstrate that the molecular reason for the absence of VvSERAT2;1 interaction with OASTL is the deletion of the last 27 amino acids contributing to the C-terminal tail. Firstly, the almost identical VvSERAT2;2 (74% sequence identity) protein differs only significantly in this C-terminal tail from the VvSERAT2;1 protein but can interact with OASTL. Secondly, engineered deletion of the C-terminal 15 amino acids in recombinant AtSERAT2;2-ΔC15 results in loss of OASTL interaction, like in case of wild type VvSERAT2;1 (Wirtz et al., 2010). Thirdly, the last 20 amino acids of plant SERAT C-terminus are sufficient to bind OASTL (Feldman-Salit et al., 2012), which, fourthly, is in full-agreement with co-crystallization of the SERAT C-terminal tail in the active site of OASTL (Francois et al., 2006). In Arabidopsis and in grapevine, CSC formation is the main trigger for regulation of SERAT2;2 activity in response to sulfide supply (see above). Astonishingly, this important regulatory element is missing in grapevine chloroplasts, which are the exclusive source of sulfide for the cell (Khan et al., 2010). Presumably, in order to compensate for this absent regulation, grapevine acquired significant transcriptional control of *VvSERAT2;1* in response to sulfate supply. The strong up-regulation of *VvSERAT2;1* transcription in response to sulfate starvation seems to be an unique feature in grapevine. Sulfate starvation did not affect transcription of AtSERAT2;1 in leaves and had only minor impact of AtSERAT2;1 transcription in roots. If the 30% decrease of *AtSERAT2;1* transcript observed in sulfate deprived roots results in significant change of AtSERAT protein remains elusive (Kawashima et al., 2005).

Transcriptional control of *VvSERAT2;1* has been also observed in response to high-light and to greater extend to drought stress. Indeed, drought stress requires sophisticated coordination of cysteine synthesis for production of the drought stress hormone, ABA (Cao et al., 2014), sulfur containing osmoprotectants (reviewed in Chan et al., 2013) and the drought-stress related retrograde signal, 3′-phosphoadenosine 5′-phosphate, which also controls part of high-light stress-response (Estavillo et al., 2011). Nevertheless, transcriptional control of *SERAT2;1* in response to drought stress has not been reported in Arabidopsis, but might be anticipated, since drought stresses is accompanied by formation of reactive oxygen species, which are known to induce transcription of *AtSERAT2;1* and *AtSERAT2;2* (Lehmann et al., 2009). Furthermore, in a catalase2-deficient mutant (*cat2*), specifically *AtSERAT2;1* is transcriptionally induced upon transfer of *cat2* from high CO_2_ to ambient air, presumably to provide more OAS for efficient synthesis of glutathione in plastids, which is needed for detoxification of reactive oxygen species (ROS) by the ascorbate/glutathione cycle in the cytosol, the plastids and the mitochondria (Wachter et al., 2005; Queval et al., 2009; Maughan et al., 2010).

Interestingly, transcriptional regulation of *SERAT* genes in response to high-light stress (Speiser et al., 2015), which also cause ROS formation, is accompanied in Arabidopsis by interaction of plastidic AtSERAT2;1 with the cyclophilin, CYP20-3 (Dominguez-Solis et al., 2008). Interaction of CYP20-3 with AtSERAT2;1 is supposed to promote formation of the plastidic CSC (Dominguez-Solis et al., 2008) and is triggered by the jasmonate precursor, (+)-12-oxo-phytodienoic acid (Park et al., 2013). This would allow hormonal control of CSC formation in plastids of Arabidopsis and would place the regulation of cysteine synthesis in plastids right in the middle of the high light and presumably also the drought stress response of plants. A note of caution must be added to the hypothesis that interaction of AtSERAT2;1 with CYP20-3 promotes CSC formation, since surface plasmon resonance and isothermal titration studies of bacterial and plant full length SERATs or its C-terminal tails demonstrate that the SERAT-OASTL interaction occurs fast and spontaneous in the absence of any molecular chaperone (Berkowitz et al., 2002; Francois et al., 2006; Zhao et al., 2006; Kumaran and Jez, 2007; Wirtz et al., 2010). However, the fact that grapevine can adapt to high light conditions unambiguously demonstrate significant differences between Arabidopsis and grapevine regarding the importance of CSC formation in plastids upon high light stress.

Our study demonstrate that many feature of the SERAT protein family, like distribution of SERATs in all sub-cellular compartments with own protein biosynthesis and the regulation of the mitochondrial SERAT activity by CSC formation are in principle conserved between grapevine and Arabidopsis. In contrast, the plastidic SERAT activity was fundamentally different regulated in grapevine when compared to Arabidopsis. This differences in regulation include the transcriptional induction of *VvSERAT2;1* in response to sulfate deficiency, the insensitivity of VvSERAT2;1 to cysteine and the inability of VvSERAT2;1 to interact with OASTL. Consequently, species specific differences in regulation of overall cellular cysteine biosynthesis must be anticipated in vascular land plants, which put a note of caution to the concept of transferring scientific findings made in the model plant Arabidopsis to crop plants.

## Materials and methods

### Biological material and growth condition

Cell suspensions of *V. vinifera* cv Touriga Nacional were obtained by adapting to liquid culture *callus* material maintained in the dark at 25°C, as described in Jackson et al. (2001). Approximately 4 g *callus* tissue were dispersed in 50 mL of liquid medium containing MS (Murashige and Skoog, 1962) basal salts supplemented with 2.5 μM 2,4-D (2,4-dichlorophenoxy-acetic acid), 5 g L^−1^polyvinylpyrrolidone −40T, 20 g L^−1^, sucrose and 1 μM kinetin. The cultures were grown in 250 mL flasks on a rotary shaker at 100 rpm, in the dark, at 25°C and subcultured weekly by dilution of 25 mL culture into 25 mL of new medium.

Two sulfate treatments were applied: full sulfate (+S, 1.5 mM SO^2−^_4_) and sulfate depleted (−S, 50 μM SO^2−^_4_), after 2 weekly cycles in +S conditions. Commercial MS (Duchefa Biochemie, Haarlem, NL) was used for +S experiments while for −S a modified MS medium where sulfate salts were substituted for chloride salts was prepared.

*Vitis vinifera* L. Touriga Nacional shoots were obtained as described in Rocheta et al. (2014) and Coito et al. (2012). Cuttings pruned in the field were disinfected with fungicide (Benlate, 2%), kept at 4°C for 2 months and rooted in the dark in complete nutrient solution (Rhue et al., 1978) diluted in distilled water (10:1, V:V). Rooted cuttings were transferred to 3 L pots filled with sterilized soil and placed in the growth room. Growth conditions were light intensity 200 μmol m^−2^ s^−1^, 16 h light/8 h dark, 25°C at day/23°C at night, and watering with nutrient solution when necessary. The potted plants were subjected to heat stress (T), drought (D) and high-light stress (H) as described in Rocheta et al. (2014) and Coito et al. (2012). The individual stresses were applied to 50–60 cm grapevine shoots, T was considered 1 h at 42°C; D when the predawn leaf water potential (Ψw) was—0.9 MPa with Ψw measured with a pressure chamber (Model 600, PMS Instruments Company, Albany, OR). H corresponds to light intensity at 1000 μmol m^−2^ s^−1^ for 1 h.

### General cloning

Standard molecular biology technologies, such as growth of bacteria, plasmid isolation, PCR product purification, and PCR were performed as described by Sambrook et al. (1989) according to good laboratory practices standards.

### RNA extraction and cDNA synthesis

Total RNA was isolated from *Vitis vinifera* cv Touriga Nacional cell cultures with the RNeasy Plant MiniKit (Qiagen, Hilden, Germany) and the RNA samples treated with DNaseI10 according to the manufacturer protocol (Qiagen, Hilden, Germany). Reverse transcription was carried out with Superscript III RNase H-reverse transcriptase (Invitrogen, Carlsbad, CA) priming with oligod(T)_20_ according to the manufacturer's recommendations. Total RNA from *V. vinifera* leaves was extracted with the RNA Plant Total RNA Kit (Sigma-Aldrich, Inc) following the manufacturer's instructions and the RNA samples treated with RQ1 RNase-Free DNase (Promega, Madison, WI). cDNA was synthesized using RevertAid Reverse Transcriptase (Fermentas Life Science, Helsingborg, Sweden) according to the manufacturer's recommendations.

### Identification of serine acetyltransferase family in *Vitis vinifera*

*In silico* analysis of SERAT conserved domains was carried out at NCBI together with the blastp analysis (http://www.ncbi.nlm.nih.gov/Structure/cdd/cdd.shtml) as described by Marchler-Bauer et al. (2009). At ExPaSy, PROSITE was used to scan the sequences for conserved motifs (http://www.expasy.ch/tools/scanprosite/).

### 5′ and 3′ -RACE analysis of VvSERAT

The 5′ and 3′ - regions of *VvSERAT2;1* and *VvSERAT2;2* transcripts were cloned by the RML-RACE technique using the FirstChoice® RLM (RNA ligase-mediated)-RACE Kit (Ambion) for amplification of full-length cDNAs. Starting from cell culture total RNA isolated as described above, all steps were performed according to the manufacturer's protocol. All amplification products were cloned into the pMOSBlue (GE Healthcare Europe GmbH) and sequenced from both ends (STAB Vida, Oeiras, Portugal), using vector-specific primers.

### Prediction of subcellular localization

We conducted an analysis in three specialized software: MultiLoc (Hoglund et al., 2006), TargetP1.1 (Emanuelsson et al., 2000) and ChloroP1-1 (Emanuelsson et al., 1999).

### Subcellular localization by fusion protein with GFP

All constructs for transient transformation were cloned into the vector pFF19 (Timmermans et al., 1990) for expression under the control of an enhanced 35S promoter. The complete ORF of each sequence was PCR amplified with specific forward primers incorporating the *Bam*HI restriction site and the reverse primers adding the *Sal*I restriction site (Supplemental Table 2). The different cDNA sequences were cloned into the *Bam*HI/*Sal*I sites of pFF19-GFP. All constructions were sequenced from both ends (STAB Vida, Oeiras, Portugal), using vector-specific primers.

The plasmid pFF19-GFP, without any fusion protein was used as a control for localization of GFP in the cytosol. The transit peptide (first 52 amino acids) of the *A. thaliana* SHMT1 (At4g37930) was fused to GFP and used as a control for mitochondrial localization. For plastidic localization the transit peptide sequence (first 36 amino acids) from ribulose-1,5-bisphosphate carboxylase small subunit polypeptide of *Pisum sativum* fused to RFP was used, in the pFF19 the EGFP was replaced by RFP (constructs provided by A. Watcher, Heidelberg, Institute for Plant Sciences, Germany).

*V. vinifera* protoplasts were obtained by incubating the cells in TEX Buffer (B5 salts; 2.5 mM 2-(*N*-morpholino)ethanesulfonic acid (MES); 5.1 mM CaCl_2_ 2H_2_O; 3.1 mM; 0.4 M sucrose; pH7 with KOH; 0.2% Macerozyme R10; 0.4% Cellulase R10), for 18 h in the dark, at 25°C. The protoplasts suspension was centrifuged for 15 min at 80 *g* at room temperature in a swing-out rotor. Living protoplasts in electroporation buffer (0.4 M Sucrose; 10 mM 4-(2-hydroxyethyl)-1-piperazineethanesulfonic acid, HEPES; 80 mM KCl; 5 mM CaCl_2_; pH 7.2 with KOH) were centrifuged twice in the same conditions. 500 μL of re-suspended protoplasts were pipetted gently into a 1 mL electroporation cuvette and 1–10 μg of plasmid diluted in 100 μL of electroporation buffer were added. The protoplasts were electroporated using 1000 μF and 130 V. After 30 min, the cuvettes were rinsed twice with 1 mL of TEX Buffer and the obtained suspension maintained in Petri dishes for 24–48 h, in the dark.

GFP and RFP localization was visualized by confocal laser microscopy (Zeiss LSM510 META system, GFP: excitation at 488 nm and emission at 510–525 nm; RFP: excitation at 568 nm and emission at 590 nm) and epifluorescence microscope (Axioskop 2; Zeiss, Jena, Germany). All images were edited with Adobe Photoshop 6.

### Transcript quantification by real-time PCR (qPCR)

Real-time PCR was performed in 20 μL of reaction mixture composed of cDNA, 0.5 μM gene-specific primers (Supplemental Table 2) and master mix iQ SYBR Green Supermix (Bio-Rad, Hercules, CA) using an iQ5 Real Time PCR (Bio-Rad, Hercules, CA). Reactions conditions for thermal cycling were: 95°C, 3 min; then 40 cycles at 95°C 15 s, 62°C 30 s, 72°C 20 s. Relative abundance of transcripts was calculated and normalized with respect to *Act2* mRNA (GI;14133880, An et al., 1996) according to the method of Livak and Schmittgen (2001). The results were expressed in Log_2_ relative to +S conditions or the control leaves.

### Purification of recombinant VvSERAT and heterologous CSC from Escherichia coli

*VvSERAT* cDNAs were cloned into the pET28a vector (Novagen, Darmstadt) for expression of recombinant protein in fusion with a N-terminal His-tag. A truncated version of the sequences, including the stop codon, was PCR amplified with specific forward primers incorporating the *Bam*HI restriction site and the reverse primers adding the *Xho*I restriction site (Supplemental Table 2).

After expression of VvSERAT recombinant proteins in *Eschericia coli* according to the manufacturers' guidelines, proteins were isolated in extraction buffer (10 mM Tris-HCl pH 8, 0.25 M sodium chloride, 20 mM Imidazole, 1 mM PMSF) and the His-VvSERATs were purified by immobilized metal affinity chromatography (Invitrogen, Germany). The immobilized VvSERATs were washed by application of 10 mL washing buffer (50 mM Tris-HCl pH 8, 0.25 M sodium chloride, 80 mM Imidazole), and 10 mL of washing buffer supplemented with 10 mM OAS to remove bacterial OASTL (CysK). His-tagged VvSERATs were eluted with 5 ml of elution buffer (50 mM Tris-HCl pH 8, 0.25 M sodium chloride, 400 mM Imidazole).

In order to test potential interaction of VvSERATs with plant OASTLs, crude extracts of *E. coli* cells expressing AtOASTL-B (Wirtz et al., 2004) were incubated with column-immobilized VvSERATs for 2 h at 20°C prior elution of His-tagged SERAT. The canonical formation of VvSERATs with AtOASTL-B was verified by on column dissociation of the heterologous CSC with 10 mM OAS dissolved in washing buffer.

Protein was determined according to Bradford (1976) using a commercial kit (Bio-Rad), against a standard curve prepared with bovine serum albumin.

### Biochemical characterization of recombinant VvSERATs

For determination of the cysteine feedback sensitivity (inhibition constant, IC_50_), enzymatic activities of VvSERATs (10–30 pmol) were tested in presence of up to 5 mM cysteine according to Wirtz et al. (2001). The impact of CSC formation on VvSERAT activity was determined after application four-fold molar excess of AtOASTL-B. In addition, SERAT activity of VvSERATs was independently confirmed by coupling of SERAT to OASTL activity as described in Wirtz et al. (2004). Efficient coupling of SERAT to OASTL activity was achieved by supplementation of 2 U purified OASTL.

Interaction between VvSERAT and OASTL-B was tested by inhibition OASTL activity upon heterologous CSC formation. AtOASTL-B (0.2–1 pmol) was incubated for 10 min at 20°C in absence (control) or presence of 4-fold excess of VvSERATs to allow CSC formation. Enzymatic activity of OASTL was determined according to Gaitonde (1967).

### Quantification of thiols and OAS

Hydrophilic metabolites were extracted from cell culture and leaves of *V. vinifera* plants according to Wirtz and Hell (2003). Thiols and amino acids were quantified after derivatization with Thiolyte™ (Calbiochem, Germany) or AccQ-Tag reagent (Waters, Germany), respectively. The derivatization procedure and separation of thiol derivatives were performed as described in Wirtz et al. (2004) by using the same HPLC system.

### Statistical analyses

One and Two-Way ANOVA were used for statistical evaluation of the results. When the *p*-value of the Two Way ANOVA was lower than 0.05 means were compared through Tukey's multiple comparison tests and statistically significant differences were accepted for *p* value lower than 0.05.

## Author contributions

Conceived and designed the experiments: ST, MW, JB. Performed the experiments: ST, MW, MB. Analyzed the data: ST, MW. Contributed reagents/materials/analysis tools: SA, MW, JB, RH. Wrote the paper: ST, MW. Revised the manuscript: SA, RH. Steered the whole study: SA, RH.

### Conflict of interest statement

The authors declare that the research was conducted in the absence of any commercial or financial relationships that could be construed as a potential conflict of interest.
