# Supplementary material for: Characterization of the serine acetyltransferase gene family of Vitis vinifera uncovers differences in regulation of OAS synthesis in woody plants
Source: Front Plant Sci. 2015 Feb 17;6:74. doi: 10.3389/fpls.2015.00074 (PMC4330696; doi:10.3389/fpls.2015.00074)
Supplement: Supplementary file 1 [file Table1.DOCX]

**Table 1. Identification of VvSERAT sequences**

| **Sequence** | **Chromosome** | **Genome** | **LOC** | **mRNA** | **Protein** |
| --- | --- | --- | --- | --- | --- |
| *VvSERAT1;1* | 11 | NW_003724068 | LOC100264650 | XM_002282514 | XP_002282550 |
| *VvSERAT2;1* | Unplaced | [NW_003724213](http://www.ncbi.nlm.nih.gov/nuccore/NW_003724213?report=graph) | LOC100260128 | XM_002270508 | XP_002270544 |
| *VvSERAT2;2* | 19 | [NW_003724146](http://www.ncbi.nlm.nih.gov/nuccore/NW_003724146?report=graph) | - | KP074964 |  |
| *VvSERAT3;1* | 4 | NW_003724017 | - | KP074965 |  |

- Not applicable

.
