# Supplementary material for: Characterization of the serine acetyltransferase gene family of Vitis vinifera uncovers differences in regulation of OAS synthesis in woody plants
Source: Front Plant Sci. 2015 Feb 17;6:74. doi: 10.3389/fpls.2015.00074 (PMC4330696; doi:10.3389/fpls.2015.00074)
Supplement: Supplementary file 2 [file Table2.DOCX]

**Table 2**  – Primer sequences used in the characterization of SERAT genes from *Vitis vinifera*.

| **Sequence Name** | **Accession Number*** | **Primer (5’ – 3’)** | |
| --- | --- | --- | --- |
| *Reporter Gene Constructs* | | | |
| VvSERAT1;1 | XM_002282514 | Fw | **GGATCC**ATGAACGGAAACCTCCATCG |
|  |  | Rv | **GTCGAC**GATGATATAATCTGACCACTC |
| VvSERAT2;1 | XM_002270508 | Fw | **GGATCC**ATGGCAGCTTGTATCCACAAC |
|  |  | Rv | **GTCGAC**GGATTTAAGGCCAACCAATC |
| VvSERAT2;2 | KP074964 | Fw | **GGATCC**ATGAAACTTCAGGCGACATC |
|  |  | Rv | **GGATCC**ATGGCAGCTTGTATCCACAAC |
| VvSERAT3;1 | KP074965 | Fw | **GGATCC**ATGCTTTGATCAGCTTCTTT |
|  |  | Rv | **GTCGAC**GATGTGGATGAAATGATTTTG |
| *Real-time PCR* | | | |
| VvSERAT1;1 | XM_002282514 | Fw | TCGCGAGTTACCTCTACTCAACGAT |
|  |  | Rv | TCACGCATATGATTGGCGCGTAGAT |
| VvSERAT2;1 | XM_002270508 | Fw | TGCAGATCATCAATGCCAGATA |
|  |  | Rv | TTCAGCCACAGAACATCATCTT |
| VvSERAT2;2 | KP074964 | Fw | GCATAAGCTACGTGCAGTGTCT |
|  |  | Rv | ATCATCTCCAATCACCGCTGTC |
| VvSERAT3;1 | KP074965 | Fw | GGGAGATGTTTCGCTTGAAAT |
|  |  | Rv | ATACTGGCATACAAGAAGCTAC |
| *5’ -RACE analysis* | | | |
| VvSERAT2;1 | XM_002270508 | Rv | GTACTTGGACAAGATGGGCTCTTGC |
| VvSERAT2;2 | KP074964 | Rv | TCAATACAAGCAGCCATGAAAAGG |
| *3’ -RACE analysis* | | | |
| VvSERAT2;1 | XM_002270508 | Fw | TGCAGATCATCAATGCCAGATA |
| VvSERAT2;2 | KP074964 | Fw | TAGGCATCCGAAGCTTGGCGATG |
|  | | | |
| *Expression of recombinant proteins* | | | |
| VvSERAT1;1 | XM_002282514 | Fw | TCAA**GGATCC**TGGCTTTGGACTCAGATCA |
|  |  | Rv | TCGG**CTCGAG**TCAGATGATATAATCTGACC |
| VvSERAT2;1 | XM_002270508 | Fw | ATC**GGATCC**GATGTTCTGTGGCTGAAG |
|  |  | Rv | CGT**CTCGAG**TTAGGATTTAAGGCCAACCA |
| VvSERAT2;2 | KP074964 | Fw | TAT**GGATCC**GGAGCTGATTTGTGGCTGAA |
|  |  | Rv | CGGC**CTCGAG**TTAAATAACATAATCAGACC |
| VvSERAT3;1 | KP074965 | Fw | ATTC**GGATCC**TATTGTGGGTGACCCGATCT |
|  |  | Rv | CTG**CTCGAG**TTAGATGTGGATGAAATGAT |

Note that primers used for reporter gene constructs and expression of recombinant proteins contained restriction sites (in bold), *Bam*HI/*Sal*I and *Bam*HI *Xho*I, respectively.

*Accession number from NCBI database
