# Supplementary material for: Characterization of the serine acetyltransferase gene family of Vitis vinifera uncovers differences in regulation of OAS synthesis in woody plants
Source: Front Plant Sci. 2015 Feb 17;6:74. doi: 10.3389/fpls.2015.00074 (PMC4330696; doi:10.3389/fpls.2015.00074)

S1. Alignment of putative SERAT proteins sequences from *Vitis vinifera* and SERAT proteins from *Arabidopsis thaliana* , showing the canonical distribution into three groups.

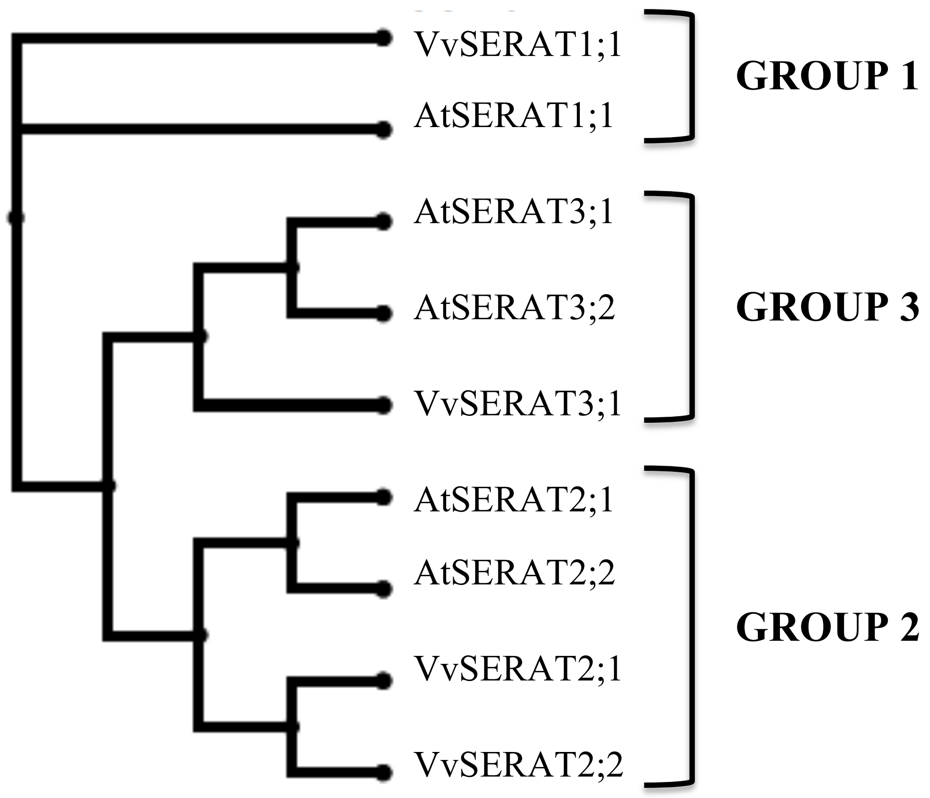

Supplement: Supplementary file 3 [file Presentation1.PDF]
