# Supplementary material for: Characterization of the serine acetyltransferase gene family of Vitis vinifera uncovers differences in regulation of OAS synthesis in woody plants
Source: Front Plant Sci. 2015 Feb 17;6:74. doi: 10.3389/fpls.2015.00074 (PMC4330696; doi:10.3389/fpls.2015.00074)
Supplement: Supplementary file 4 [file Presentation2.PPTX]

## Slide 1
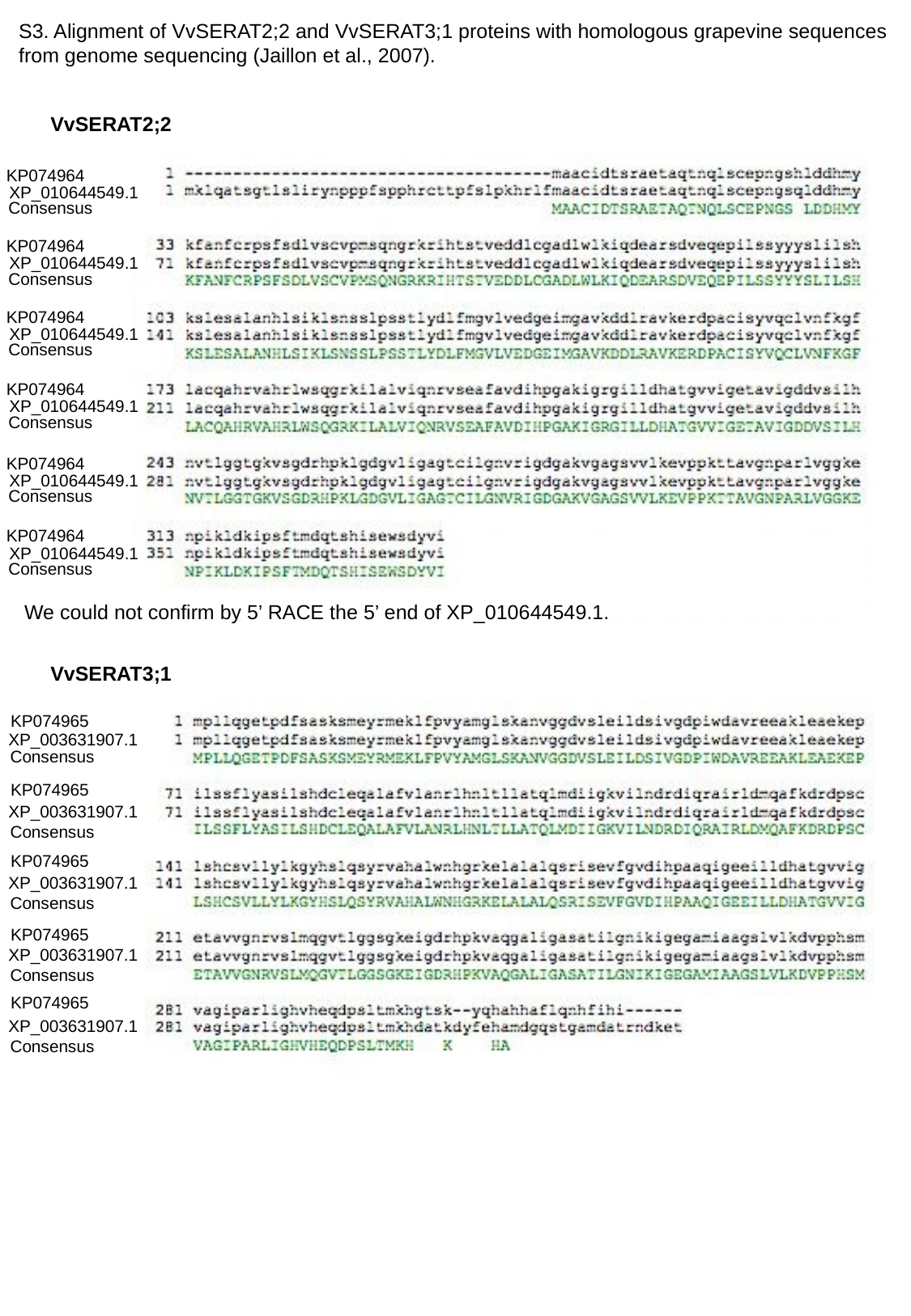

S3. Alignment of VvSERAT2;2 and VvSERAT3;1 proteins with homologous grapevine sequences from genome sequencing (Jaillon et al., 2007).
VvSERAT2;2
XP_010644549.1
KP074964
Consensus
XP_010644549.1
KP074964
Consensus
XP_010644549.1
KP074964
Consensus
XP_010644549.1
KP074964
Consensus
XP_010644549.1
KP074964
Consensus
XP_010644549.1
KP074964
Consensus
We could not confirm by 5’ RACE the 5’ end of XP_010644549.1.
VvSERAT3;1
KP074965
XP_003631907.1
Consensus
KP074965
XP_003631907.1
Consensus
KP074965
XP_003631907.1
Consensus
KP074965
XP_003631907.1
Consensus
KP074965
XP_003631907.1
Consensus
