# Supplementary figures and images for: Characterization of the serine acetyltransferase gene family of Vitis vinifera uncovers differences in regulation of OAS synthesis in woody plants
Source: Front Plant Sci. 2015 Feb 17;6:74. doi: 10.3389/fpls.2015.00074 (PMC4330696; doi:10.3389/fpls.2015.00074)

### S3. *VvSERAT* gene structure

*VvSERAT1;1* (1.25 Kbp)

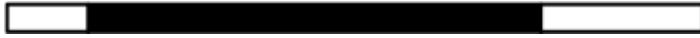

*VvSERAT2;1* (1.4 Kbp)

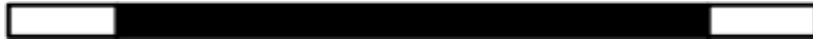

*VvSERAT2;2* (1.4 kbp)

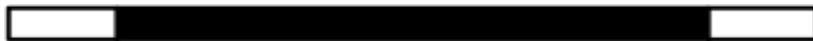

*VvSERAT3;1* (5 Kbp)

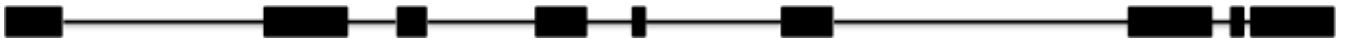

— Intron

■ Exon

□ UTR

Supplement: Supplementary file 5 [file Presentation3.PDF]
